# Gene expression levels of gamma-glutamyl hydrolase in tumor tissues may be a useful biomarker for the proper use of S-1 and tegafur-uracil/leucovorin in preoperative chemoradiotherapy for patients with rectal cancer

**DOI:** 10.1007/s00280-017-3295-8

**Published:** 2017-04-17

**Authors:** Sotaro Sadahiro, T. Suzuki, A. Tanaka, K. Okada, G. Saito, H. Miyakita, T. Ogimi, H. Nagase

**Affiliations:** 1 Department of Surgery, Tokai University School of Medicine, 143 Shimokasuya, Isehara, Kanagawa 259-1193 Japan; 2 Applied Pharmacology Lab., Taiho Pharmaceutical Co., Ltd., 224-2 Ebisuno Hiraishi, Kawauchi-cho, Tokushima, 771-0194 Japan

**Keywords:** Rectal cancer, Chemoradiotherapy, Tegafur-uracil/leucovorin, S-1, Predictive factors, Gamma-glutamyl hydrolase

## Abstract

**Purpose:**

Preoperative chemoradiotherapy (CRT) using 5-fluorouracil (5-FU)-based chemotherapy is the standard of care for rectal cancer. The effect of additional chemotherapy during the period between the completion of radiotherapy and surgery remains unclear. Predictive factors for CRT may differ between combination chemotherapy with S-1 and with tegafur-uracil/leucovorin (UFT/LV).

**Methods:**

The subjects were 54 patients with locally advanced rectal cancer who received preoperative CRT with S-1 or UFT/LV. The pathological tumor response was assessed according to the tumor regression grade (TRG). The expression levels of 18 CRT-related genes were determined using RT-PCR assay.

**Results:**

A pathological response (TRG 1-2) was observed in 23 patients (42.6%). In a multivariate logistic regression analysis for pathological response, the overall expression levels of four genes, HIF1A, MTHFD1, GGH and TYMS, were significant, and the accuracy rate of the predictive model was 83.3%. The effects of the gene expression levels of GGH on the response differed significantly according to the treatment regimen. The total pathological response rate of both high-GGH patients in the S-1 group and low-GGH patients in the UFT/LV group was 58.3%.

**Conclusion:**

Additional treatment with 5-FU-based chemotherapy during the interval between radiotherapy and surgery is not beneficial in patients who have received 5-FU-based CRT. The expression levels of four genes, HIF1A, MTHFD1, GGH and TYMS, in tumor tissues can predict the response to preoperative CRT including either S-1 or UFT/LV. In particular, the gene expression level of GGH in tumor tissues may be a useful biomarker for the appropriate use of S-1 and UFT/LV in CRT.

## Introduction

Multidisciplinary treatment including preoperative radiotherapy or chemoradiotherapy (CRT) significantly decreases local recurrence in patients with locally advanced rectal cancer and has been established as a standard treatment [1–4]. The histological response to preoperative CRT is closely related to the oncologic outcome. Disease-free survival and overall survival are significantly better in patients with histologic complete regression or with tumor down-staging than in patients without such findings [5–8].

5-Fluorouracil (5-FU)-based chemotherapy has been commonly used in combination with radiotherapy, and 5-FU seems to have radiosensitizing properties. Approximately, 20% of patients treated with neoadjuvant 5-FU-based CRT have been reported to show a pathological complete response (pCR) [8, 9]. An additional neoadjuvant administration of mFOLFOX6 (5-FU, leucovorin (LV), and oxaliplatin) during the period between the completion of radiotherapy and surgery has been reported to have the potential to increase the rate of pCR [10].

Both S-1 and tegafur-uracil (UFT) are 5-FU-based oral drugs. UFT is commonly used in combination with LV to enhance the 5-FU-induced inhibition of thymidine synthase (TYMS) [11–13]. Combination chemotherapy with oral UFT and oral LV reportedly has a comparable therapeutic efficacy to intravenous 5-FU and LV [14, 15]. S-1 contains CDHP (5-chloro-2,4-dihydroxypyridine), which is a more potent inhibitor of dihydropyrimidine dehydrogenase (DPYD) than uracil in UFT, resulting in the maintenance of a high concentration of 5-FU in the tumor tissues. Therefore, S-1 has a stronger antitumor effect than UFT and is commonly used without LV. We previously reported the efficacy and safety of oral UFT/LV therapy for the long-term treatment of patients with colon cancer [16] and of CRT including UFT or S-1 in patients with rectal cancer [17–21].

The first objective of this study was to investigate the effect of additional oral UFT/LV or S-1 therapy during the period between the completion of radiotherapy and surgery on the pCR and histological response.

We previously reported that the tumor expression levels of folate-related genes, such as folylpolyglutamate synthase (FPGS) and gamma-glutamyl hydrolase (GGH), and of 5-FU-related genes, such as thymidine phosphorylase (TYMP), thymidylate synthetase (TYMS), and dihydropyrimidine dehydrogenase (DPYD), are closely correlated with the response to preoperative chemotherapy or chemoradiotherapy including UFT or S-1 [8, 22–24]. Since the most substantial difference between these two 5-FU-based chemotherapies, i.e., UFT/LV and S-1, is the presence/absence of LV, the predictive factors may differ when UFT/LV and S-1 are used for CRT.

As a second objective of this study, we investigated the association between gene expression levels in tumors before CRT and the pathological response to CRT to establish a useful biomarker for the proper use of S-1 and UFT/LV.

## Patients and methods

### Patients

Sixty consecutive patients with clinical stage II or stage III histologically confirmed adenocarcinoma of the middle or lower third of the rectum who were treated at Tokai University Hospital between 2013 and 2016 were enrolled in this study. Six patients were excluded because of the unavailability of biopsy specimens obtained before CRT, the unavailability of resected specimens, or the refusal of the patient to undergo surgery. Therefore, data for 54 patients were used as the analysis set. The initial evaluation included a digital examination of the rectum, chest radiography, colonoscopy, barium enema, computed tomography of the abdomen and pelvis, endorectal ultrasonography, and magnetic resonance imaging (MRI) of the pelvis. Patients were randomly assigned to one of the four treatment groups. The study investigators and patients were not blinded to the treatment assignments.

This study was conducted with the approval of the Ethics Committees of Tokai University School of Medicine (15R-153) and Taiho Pharmaceutical Co., Ltd. (SN15-011). All the patients provided written informed consent. The patient characteristics are shown in Table 1.

**Table 1:** Patient characteristics and association between clinical parameters and pathological tumor response or tumor shrinkage

Parameter	Total	pCR			Responder			Tumor shrinkage (%)			
					(TRG 1 and 2)			Ba enema		MRI	
	*n*	*n*	(%)	*P* value	*n*	(%)	*P* value	Mean ± SD	*P* value	Mean ± SD	*P* value
All patients	54	8	14.8		23	42.6		51.4 ± 17.4		73.1 ± 15.4	
Sex											
Female	15	2	13.3		7	46.7		55.2 ± 18.3		73.4 ± 13.1	
Male	39	6	15.4	1.0000	16	41.0	0.7648	50.0 ± 17.0	0.3265	73.0 ± 16.2	0.9339
Age (in years)											
>65	29	6	20.7		16	55.2		52.6 ± 18.5		75.0 ± 13.8	
≤65	25	2	8.0	0.2621	7	28.0	0.0568	50.1 ± 16.2	0.6124	71.1 ± 17.0	0.3630
Primary tumor site											
Middle rectum (Ra)	18	3	16.7		7	38.9		52.8 ± 16.6		71.2 ± 18.2	
Lower rectum (Rb)	36	5	13.9	1.0000	16	44.4	0.7756	50.7 ± 17.9	0.6777	74.0 ± 14.0	0.5418
Histological type											
Well	39	6	15.4		18	46.2		50.7 ± 15.1		74.4 ± 15.0	
Moderate	15	2	13.3	1.0000	5	33.3	0.5414	53.4 ± 22.7	0.6090	69.9 ± 16.3	0.3356
Regimen											
S-1	27	4	14.8		10	37.0		50.8 ± 19.5		75.6 ± 13.4	
UFT/LV	27	4	14.8	1.0000	13	48.1	0.5826	52.0 ± 15.3	0.7987	70.6 ± 17.1	0.2382
Period of chemotherapy											
S-1 for 5 weeks	14	3	21.4		6	42.9		45.1 ± 21.7		74.9 ± 13.9	
S-1 for 11 weeks	13	1	7.7	0.5956	4	30.8	0.6946	56.9 ± 15.2	0.1179	76.3 ± 13.3	0.7946
UFT/LV for 5 weeks	13	2	15.4		7	53.8		55.0 ± 14.2		72.8 ± 15.5	
UFT/LV for 10 weeks	14	2	14.3	1.0000	6	42.9	0.7064	49.3 ± 16.4	0.3427	68.4 ± 18.9	0.5237

The *P* values were calculated using the Fisher exact test for categorical data and the Student *t* test for numerical data

*pCR* pathological complete response, *TRG* tumor regression grade, *Ba enema* barium enema examination, *MRI* magnetic resonance imaging

### Treatment

**Fig. 1:**
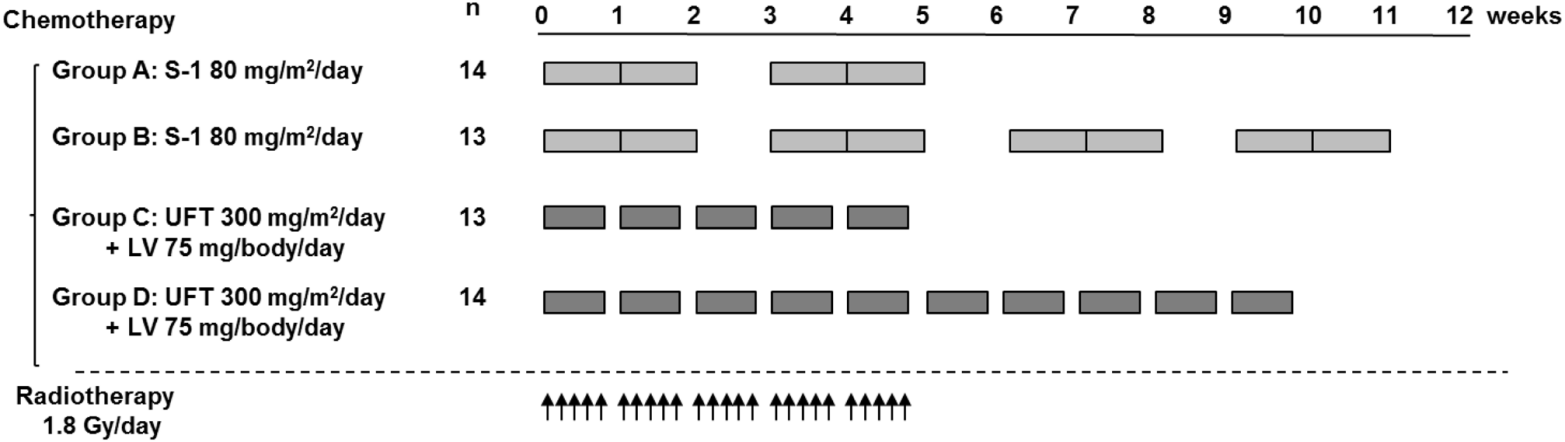
Preoperative chemoradiotherapy including S-1 or UFT in patients with rectal cancer. Oral S-1 (80 mg/m^2^) was administered daily per a 2-weeks-on/1-week-off schedule. This cycle was repeated once (group A) or three times (group B). Oral tegafur/uracil (UFT) (300 mg/m^2^) and leucovorin (LV) (75 mg/body) were administered daily per a 5-days-on/2-days-off schedule for 5 weeks (group C) or for 10 weeks (group D). Irradiation was performed once (1.8 Gy) daily per a 5-days-on/2-days-off schedule for 5 weeks

The treatment schedule for CRT is shown in Fig. 1. Preoperative radiotherapy was performed for all the patients using 18 MeV X-ray beams delivered by a linear accelerator (Clinac 2100 C; Varian Medical Systems, Inc., Palo Alto, CA, USA) using the four-field technique. Irradiation was performed once (1.8 Gy) daily to a total dose of 45 Gy. Surgery was performed 7–9 weeks (median, 59 days) after the completion of radiotherapy. Patients in Group A received 5 weeks of radiotherapy with oral S-1 (80 mg/m^2^) as concomitant chemotherapy. Oral S-1 was given for 2 consecutive weeks, followed by a 1-week rest, and was then given for 2 more weeks [18]. Patients in Group B received the same chemoradiotherapy during the first 5 weeks as Group A and received an additional 4 weeks of oral S-1 chemotherapy until 11 weeks, according to the same dosage schedule. Patients in Group C received 5 weeks of radiotherapy with oral UFT (300 mg/m^2^) and oral LV (75 mg/body) as concomitant chemotherapy. Oral UFT and LV were given for 5 days, followed by a 2-day rest. This cycle was repeated for 5 weeks during radiotherapy. Patients in Group D received the same chemoradiotherapy during the first 5 weeks as Group C and received an additional 5 weeks of oral UFT/LV chemotherapy until 10 weeks. During the protocol treatments, clinical findings and laboratory data were evaluated every week during the first 5 weeks. After the completion of the radiotherapy, the blood chemistry findings of the patients were examined every 2 weeks until surgery.

### Tissue sampling

A colonoscopy was performed to obtain biopsy specimens before CRT. We obtained six biopsy specimens from each patient. All the biopsy samples were immediately immersed in RNAlater solution (Thermo Fisher Scientific, Waltham, MA, USA) and incubated overnight at 4 °C. Then, the tissues were removed from the RNAlater solution and stored at −80 °C.

### Evaluation of antitumor effectiveness

The antitumor effectiveness was evaluated based on the histologic regression observed in the resected specimen. Histologic regression was classified according to the tumor regression grade (TRG) [25]. TRG was classified as Grade 1 (complete regression), Grade 2 (presence of rare residual cancer cells), Grade 3 (increased number of residual cancer cells), Grade 4 (residual cancer outgrowing fibrosis), or Grade 5 (absence of regression change). A patient with a TRG of 1 or 2 was defined as a responder. Barium enema and MRI have been used to evaluate tumor shrinkage after CRT [26, 27]. A barium enema examination can evaluate 2-dimensional changes, consistent with the response evaluation criteria in solid tumors [28], whereas MRI-based volumetry can assess 3-dimensional changes. In the present study, both evaluation methods were used in 53 patients and evaluation based only on a barium enema examination was performed for one patient. The double-contrast barium enema examinations and magnetic response volumetry studies were performed before CRT and immediately before surgery. A 1.5-Tesla MRI system with a surface coil was used. Before MRI, colonic irrigation was performed, and the barium was then infused. Cross-sectional areas were measured on axial T2 images. The degree of tumor shrinkage on the barium enema examination was calculated by measuring the tumor along the major axis (length along the long axis of the bowel) on lateral views. Each measurement was corrected by the diameter of the first sacrum [26]. The following formula was used to calculate tumor shrinkage: Degree of tumor shrinkage (%) = [1 − *B* × (*C*/*D*)/*A*] × 100 (%), where *A* = length of tumor before CRT; *B* = length of tumor immediately before surgery; *C* = diameter of the first sacral vertebral body before CRT; and *D* = diameter of the first sacral vertebral body immediately before surgery. The degree of tumor shrinkage on MRI was calculated according to the following formula: Tumor shrinkage rate (%) = (tumor volume before CRT − tumor volume after CRT)/tumor volume before CRT × 100 (%).

### Gene expression analysis

The mRNA expressions of 5-FU-related enzymes [six genes: dihydropyrimidine dehydrogenase (DPYD), ribonucleotide reductase M1 (RRM1), thymidine kinase 1 soluble (TK1), thymidine phosphorylase (TYMP), thymidylate synthetase (TYMS), and uridine monophosphate synthetase (UMPS)], of reduced folate-related enzymes [eight genes: 5-aminoimidazole-4-carboxamide ribonucleotide formyltransferase/IMP cyclohydrolase (ATIC), dihydrofolate reductase (DHFR), folylpolyglutamate synthase (FPGS), phosphoribosylglycinamide formyltransferase (GART), gamma-glutamyl hydrolase (GGH), methylenetetrahydrofolate dehydrogenase 1 (MTHFD1), methylenetetrahydrofolate reductase (MTHFR), and 5,10-methenyltetrahydrofolate synthetase (MTHFS)], and of radiation-related enzymes [four genes: cyclin-dependent kinase inhibitor 1A (CDKN1A), hypoxia inducible factor 1 alpha subunit (HIF1A), tumor protein p53 (TP53), and vascular endothelial growth factor A (VEGFA)] were quantitatively evaluated using a RT-PCR assay, as described below. Total RNA was isolated from the tissue using the RNeasy mini kit (Qiagen, Valencia, CA, USA) and reverse transcribed using a high-capacity cDNA reverse transcription kit (Thermo Fisher Scientific). A real-time reverse transcription polymerase chain reaction (RT-PCR) was performed using an ABI PRISM 7900HT sequence detection system (Thermo Fisher Scientific) and a real-time RT-PCR array (TaqMan Array; Thermo Fisher Scientific), which included duplicated wells of a reference gene (Assay ID: beta-actin [ACTB] Hs99999903_m1) and 18 target genes (Assay ID: DPYD Hs00559279_m1, RRM1 Hs01040698_m1, TK1 Hs00177406_m1, TYMP Hs00157317_m1, TYMS Hs00426586_m1, UMPS Hs00165978_m1, ATIC Hs00269671_m1, DHFR Hs00758822_s1, FPGS Hs00191956_m1, GART Hs00531926_m1, GGH Hs00608257_m1, MTHFD1 Hs01068263_m1, MTHFR Hs00195560_m1, MTHFS Hs00197574_m1, CDKN1A Hs00355782_m1, HIF1A Hs00153153_m1, TP53 Hs01034249_m1, and VEGFA Hs00900055_m1). The gene expression levels were normalized to the reference gene, ACTB [29, 30]. The relative gene expression levels were calculated using the delta threshold cycle (Ct) method according to the formula shown below. The expression levels of the target genes were expressed as 2^−(delta *Ct*)^ × 1000 to simplify the calculation.

$$\begin{gathered} {\text{Expression level of target gene}}={2^{ - ({\text{delta }}Ct)}} \times 1000. \hfill \\ {\text{Delta }}Ct=\left( {Ct{\text{ of target gene}}} \right)-\left( {Ct{\text{ of beta-actin}}} \right). \hfill \\ \end{gathered}$$

### Statistical analysis

The associations between clinical parameters (sex, age, primary tumor site, histological type, primary tumor site, regimen and period of chemotherapy) and the tumor response to CRT and tumor shrinkage were evaluated using the Fisher exact test and the Student *t* test, respectively. The associations between gene expression levels in tumor tissue before CRT and the response to CRT were evaluated using univariate and multivariate logistic regression analyses. The odds ratios were calculated as the value per change in regressor over one unit. *P* values were calculated using the Wald test. The patients were divided into low and high groups according to the gene expression levels of γ-glutamyl hydrolase (GGH) using the median as the cut-off value. The presence of an interaction between the regimen and GGH expression was assessed using a multivariate logistic regression analysis. *P* values were calculated using the Wald test. The difference in the response rates between the low and high expression groups were evaluated using the Fisher exact test. Log-transformed values of the gene expression levels were used for all the statistical analyses. JMP 9.0.2 statistical software (SAS Institute Inc., Cary, NC, USA) was used. Differences were considered significant when *P* < 0.05. All the statistical analyses were conducted with the support of the Sugimoto Data Analysis Service (Aichi, Japan).

## Results

A pathological response (TRG 1–2) and a pCR were observed in 42.6% (23/54) and 14.8% (8/54) of the total patients, respectively (Table 1). The intervals until surgery from the completion of radiation therapy in Groups A, B, C and D were 59.3 ± 4.2, 59.6 ± 6.0, 59.7 ± 4.4 and 64.6 ± 26.8 days, respectively. There were no significant differences in the intervals among the 4 groups (data not shown).

There were no significant relationships between the rate of response, pCR, or tumor shrinkage and the clinical parameters (sex, age, primary tumor site, histological type, or regimen) (Table 1). The addition of oral UFT/LV or S-1 therapy during the period between the completion of radiotherapy and surgery did not have a significant effect on the pCR and pathological response (Table 1).

**Fig. 2:**
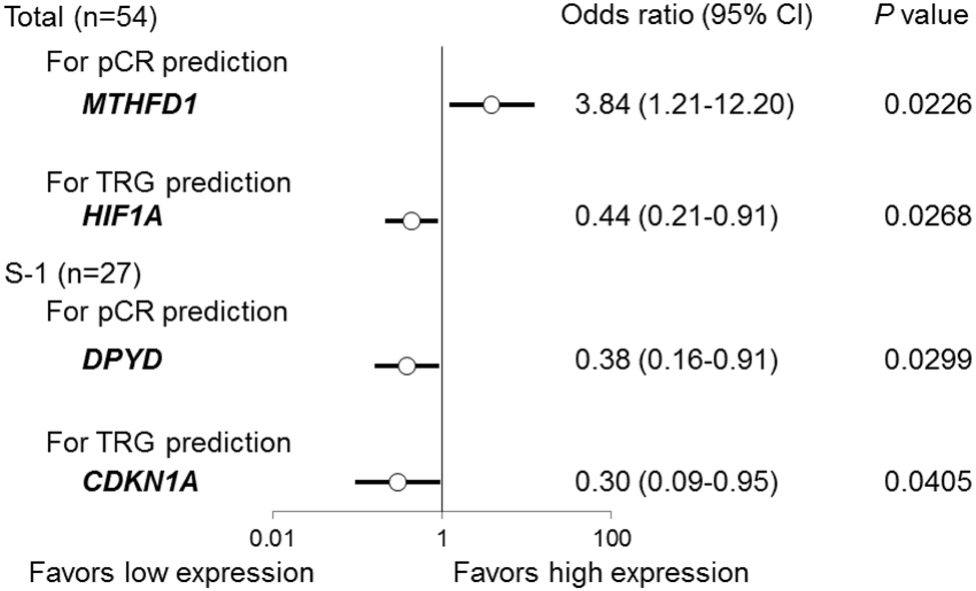
Univariate logistic regression analysis for the pathological response to CRT using gene expression levels in tumor tissues before CRT. The odds ratios are the values per change in regressor over one unit. *95% CI* 95% confidence interval. The *P* values were calculated using the Wald test. In the UFT/LV group (*n* = 27), none of the gene expressions were significantly associated with the pathological response to CRT

In a univariate logistic regression analysis performed using all the patients, the gene expression levels of MTHFD1 and HIF1A were significantly associated with the pCR and the TRG response, respectively (Fig. 2). The odds ratios of MTHFD1 for pCR prediction and of HIF1A for TRG prediction were 3.84 (1.21–12.20) and 0.44 (0.21–0.91), respectively. In the S-1 group, the gene expression levels of DPYD and CDKN1A were significantly associated with the pCR and with the TRG response, respectively (Fig. 2). The odds ratios of DPYD for pCR prediction and of CDKN1A for TRG prediction were 0.38 (0.16–0.91) and 0.30 (0.09–0.95), respectively. In the UFT/LV group, no significant association between gene expression and the pCR or the TRG response was observed.

**Fig. 3:**
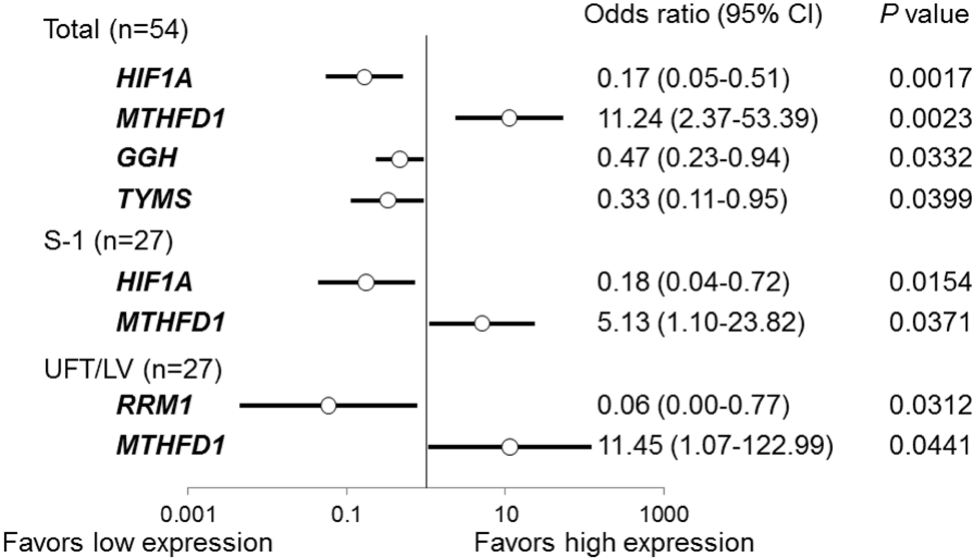
Multivariate logistic regression analysis for the pathological response (TRG) to CRT using gene expression levels in tumor tissues before CRT. The odds ratios are the values per change in regressor over one unit. *95% CI* 95% confidence interval. Variables were selected using stepwise regression. The *P* values were calculated using the Wald test

In a multivariate logistic regression analysis of the pathological response (TRG), the expression levels of four genes (HIF1A, MTHFD1, GGH and TYMS) in all the patients, of two genes (HIF1A and MTHFD1) in the S-1 group, and of two genes (RRM1 and MTHFD1) in the UFT/LV group were identified as being significant using stepwise regression (Fig. 3). The accuracy rates of the predictive models using the logistic regression technique in all the patients, in the S-1 group, and in the UFT/LV group were 83.3, 81.5, and 74.1%, respectively (Table 2).

**Table 2:** Accuracy rates of the models for TRG prediction using the logistic regression technique

Regimen	*n*	Variables (gene symbol)	Sensitivity (%)	Specificity (%)	Accuracy rate (%)
Total	54	*HIF1A*	88.9	80.6	83.3
		*MTHFD1*			
		*GGH*			
		*TYMS*			
S-1	27	*HIF1A*	69.2	92.9	81.5
		*MTHFD1*			
UFT/LV	27	*RRM1*	75.0	73.3	74.1
		*MTHFD1*			

The cut-off values were determined using the receiver operating characteristic (ROC) curve

**Fig. 4:**
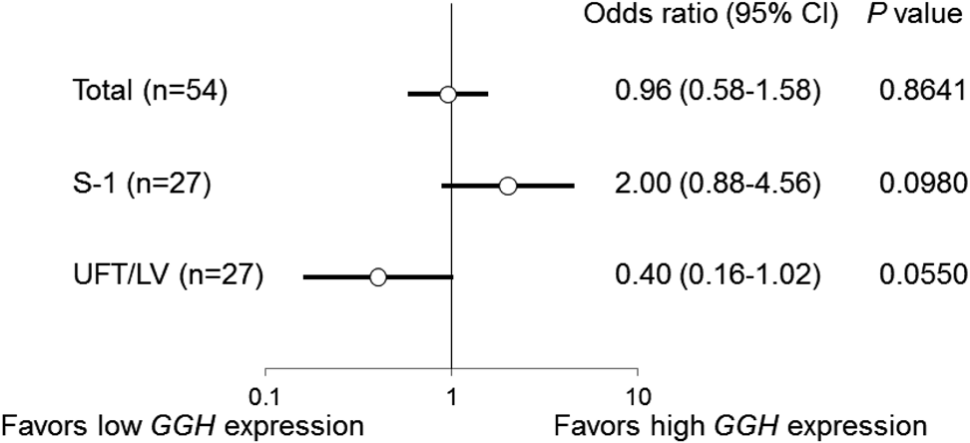
Univariate logistic regression analysis for the pathological response (TRG) to CRT using GGH gene expression levels in tumor tissues before CRT. The odds ratios are the values per change in regressor over one unit. *95% CI* 95% confidence interval. The *P* values were calculated using the Wald test

A high gene expression of GGH was associated with resistance to CRT in the UFT/LV group [odds ratio 0.40 (0.16–1.02)]. In contrast, high gene expression of GGH was associated with sensitivity to CRT in the S-1 group [odds ratio 2.00 (0.88–4.56)] (Fig. 4). In a multivariate logistic regression analysis for the pathological response (TRG), the regimen, GGH, and regimen × GGH, a significant qualitative interaction was observed between the regimen and GGH (*P* = 0.0113, data not shown). In other words, the effects of the gene expression levels of GGH on the response were differed significantly according to the regimen. The total pathological response rate of both high-GGH patients in the S-1 group and low-GGH patients in the UFT/LV group was 58.3%, which was higher than that observed for all the patients (42.6%) (Table 3).

**Table 3:** Association between pathological tumor response by TRG and GGH gene expression

Regimen	*GGH*	Total	Responder		*P* value
	High/low	*n*	*n*	(%)	
Total		54	23	42.6	
S-1	High	12	6	50.0	0.2566
	Low	15	4	26.7	
UFT/LV	High	15	5	33.3	0.1283
	Low	12	8	66.7	
S-1	High	24	14	58.3	
+					
UFT/LV	Low				

The *P* values were calculated using the Fisher exact test

The median GGH gene expression level for all the patients was used as the cut-off value

## Discussion

A longer radiation-surgery interval from the end of neoadjuvant CRT is reportedly associated with higher rates of pCR in the treatment of rectal cancer, but intervals of longer than 8 weeks were associated with higher rates of positive circumferential resection margins [31–33]. A multicenter randomized controlled trial (GRECCAR-6) showed that waiting 11 weeks after CRT did not increase the rate of pCR after surgical resection, compared with 7 weeks after CRT, and a longer waiting period was associated with higher morbidity and a more difficult surgical resection [34]. Lengthening the interval between radiation and surgery and the additional neoadjuvant administration of mFOLFOX6 reportedly increased the rate of pCR, but the proportion of patients experiencing adverse events during mFOLFOX6 treatment also increased among patients with locally advanced rectal cancer [10].

Based on these results, in this study, we adopted an interval of 8–9 weeks from the completion of radiation therapy until surgery and added two groups (Groups B and D) in which S-1 or UFT/LV was continuously administered until surgery after the completion of CRT. Then, we investigated the pathological responses in the following four groups: Group A, S-1 for 5 weeks; Group B, S-1 for 11 weeks; Group C, UFT/LV for 5 weeks; and Group D, UFT/LV for 10 weeks.

The rate of pCR among all the patients was 14.8%, and this rate remained the same for both the S-1 and UFT/LV groups (Table 1). The rate was almost the same as previously reported rates for 5-FU-based CRT in patients with rectal cancer [8, 9]. Unexpectedly, the addition of oral UFT/LV or S-1 therapy during the period between the completion of radiotherapy and surgery did not affect the pathological response rate (Table 1). These results suggest that additional 5-FU-based chemotherapy treatments during the interval between CRT and surgery are not beneficial in patients who have received 5-FU-based CRT.

Although both S-1 and UFT are 5-FU-based oral drugs, UFT is commonly used in combination with LV to enhance the effects of 5-FU, but S-1 is commonly used without LV since S-1 has a stronger antitumor effect than UFT. Therefore, the absence/presence of LV may affect predictive factors, such as folate-related genes. Actually, we previously reported that the gene expression levels of folylpolyglutamate synthase (FPGS) and gamma-glutamyl hydrolase (GGH) in tumors could predict the levels of reduced folate after LV administration in tumor tissue [23]. Moreover, we have reported that a reduction in GGH gene expression was associated with the response to UFT/LV chemotherapy in patients with colorectal cancer [24].

In this study, we investigated the association between gene expression levels in tumors before CRT and the pathological response to CRT to establish a useful biomarker for the appropriate use of S-1 and UFT/LV. Because the length of administration of S-1 or UFT/LV did not affect the pathological response rate, we analyzed the association in two groups: the S-1 group (groups A and B) and the UFT/LV group (Groups C and D).

The analysis showed that a high gene expression of GGH was associated with resistance to CRT in the UFT/LV group, but that a high gene expression of GGH was associated with sensitivity to CRT in the S-1 group (Fig. 4). Therefore, these results suggest that the gene expression level of GGH in tumor tissues may be a useful biomarker for determining which regimen, S-1 or UFT/LV, should be used for CRT. Actually, the total pathological response rate of both high-GGH patients in the S-1 group and low-GGH patients in the UFT/LV group was 58.3%, which was higher than the rate for all the patients (42.6%) (Table 3).

GGH is a lysosomal enzyme that acts as an endopeptidase and an exopeptidase to remove the terminal glutamates of the polyglutamated folates synthesized by FPGS [35, 36]. The polyglutamate forms of folate are more easily retained within cells [35]. We previously reported that the siRNA down-regulation of GGH mRNA increased both the intracellular folate level after LV treatment and the cellular sensitivity to FdUrd plus LV [37]. We also reported that the expression level of GGH was negatively correlated with the level of reduced folate in CRC tissues [23]. Therefore, the low levels of GGH mRNA might lead to an elevated folate level in tumor tissue, thereby enhancing the antitumor effect of UFT/LV chemotherapy. On the other hand, the antitumor effect of S-1 chemotherapy without LV may not be affected by the gene expression levels of GGH. However, the reason why a high gene expression level of GGH was associated with sensitivity to CRT in the S-1 group remains unknown.

In a multivariate logistic regression analysis of the pathological response (TRG) in all the patients, the expression levels of four genes, HIF1A, MTHFD1, GGH and TYMS, were significantly selected using stepwise regression (Fig. 3), and a higher accuracy rate (83.3%) for predictive models using these four genes was obtained in this study (Table 2). Therefore, the gene expression levels of four genes, HIF1A, MTHFD1, GGH and TYMS, in tumor tissues before CRT may be useful for predicting the efficacy of preoperative CRT including S-1 or UFT/LV in patients with rectal cancer. In particular, MTHFD1 was selected as a common predictor for both the S-1 and UFT/LV groups (Fig. 3), i.e. independently of the administration of LV. MTHFD1 is an enzyme with the following three functions: the ATP-dependent conversion of formate and tetrahydrofolate (FH_4_) to 10-formyltetrahydrofolate (10-CHOFH_4_) (synthetase); the interconversion of 10-CHOFH_4_ and 5,10-methenyltetrahydrofolate (5,10-CHFH_4_) (cyclohydrolase); and the NADP-dependent reduction of 5,10-CHFH_4_ to 5,10-methylenetetrahydrofolate (5,10-CH_2_FH_4_) (dehydrogenase) [38]. Tsukihara et al. recently reported that the reduction of the expression of MTHFD1 using small-interfering RNA decreased the in vitro cytotoxicity of FdUrd, the active form of 5-FU, in the human colorectal cancer cell lines DLD-1 and HCT116 [39]. These facts suggest that MTHFD1 may work as an enzyme that produces 5,10-CH_2_FH_4_, which is essential for the inhibition of TYMS by 5-FU, independently of the administration of LV in CRT that includes 5-FU-based drugs.

TYMS is one of the principle enzymes involved in DNA synthesis and is a molecular target of 5-FU [40]. An inverse relationship has been reported between TYMS expression levels and the response to 5-FU [41–43]. We have previously reported that a low level of TYMS gene expression in tumor tissues before CRT was associated with the response in rectal cancer patients receiving preoperative CRT including S-1 or UFT [8]. In accordance with these previous reports, a negative correlation between the gene expression level of TYMS and the response to CRT including S-1 or UFT/LV was observed in a multivariate analysis performed in this study.

HIF1 is an enzyme with a key role in the cellular response to hypoxia, and the alpha subunits of HIF (HIF1A) are rapidly degraded by proteasomes under normoxia, but are stabilized by hypoxia. HIF-1 affects many processes that have been shown to influence radio-responsiveness, including glycolysis, mitosis, apoptosis, and angiogenesis [44]. Toiyama et al. reported that a low gene expression level of HIF1A in pre-treatment tumor biopsies was significantly associated with a high rate of tumor regression in patients with rectal cancer treated with neoadjuvant 5-FU-based CRT [45]. In accordance with this previous report, a negative correlation between the gene expression level of HIF1A and the response to CRT including S-1 or UFT/LV was observed in a multivariate analysis performed in this study.

## Conclusions

Additional 5-FU-based chemotherapy treatments during the interval between CRT and surgery are not beneficial to patients who have received 5-FU-based CRT. The expression levels of four genes (HIF1A, MTHFD1, GGH and TYMS) in tumor tissue before CRT can predict the response to preoperative CRT including S-1 or UFT/LV. In particular, the gene expression level of GGH in tumor tissues may be a useful biomarker for determining which regimen, S-1 or UFT/LV, should be used for CRT.

## References

[1] Bosset JF, Collette L, Calais G, Mineur L, Maingon P, Radosevic-Jelic L, Daban A, Bardet E, Beny A, Ollier JC (2006). Chemotherapy with preoperative radiotherapy in rectal cancer. N Engl J Med.

[2] Gerard JP, Conroy T, Bonnetain F, Bouche O, Chapet O, Closon-Dejardin MT, Untereiner M, Leduc B, Francois E, Maurel J, Seitz JF, Buecher B, Mackiewicz R, Ducreux M, Bedenne L (2006). Preoperative radiotherapy with or without concurrent fluorouracil and leucovorin in T3-4 rectal cancers: results of FFCD 9203. J Clin Oncol.

[3] Peeters KC, Marijnen CA, Nagtegaal ID, Kranenbarg EK, Putter H, Wiggers T, Rutten H, Pahlman L, Glimelius B, Leer JW, van de Velde CJ (2007). The TME trial after a median follow-up of 6 years: increased local control but no survival benefit in irradiated patients with resectable rectal carcinoma. Ann Surg.

[4] Sauer R, Liersch T, Merkel S, Fietkau R, Hohenberger W, Hess C, Becker H, Raab HR, Villanueva MT, Witzigmann H, Wittekind C, Beissbarth T, Rodel C (2012). Preoperative versus postoperative chemoradiotherapy for locally advanced rectal cancer: results of the German CAO/ARO/AIO-94 randomized phase III trial after a median follow-up of 11 years. J Clin Oncol.

[5] de Campos-Lobato LF, Stocchi L, da Luz Moreira A, Kalady MF, Geisler D, Dietz D, Lavery IC, Remzi FH, Fazio VW (2010). Downstaging without complete pathologic response after neoadjuvant treatment improves cancer outcomes for cIII but not cII rectal cancers. Ann Surg Oncol.

[6] Maas M, Nelemans PJ, Valentini V, Das P, Rodel C, Kuo LJ, Calvo FA, Garcia-Aguilar J, Glynne-Jones R, Haustermans K, Mohiuddin M, Pucciarelli S, Small W, Suarez J, Theodoropoulos G, Biondo S, Beets-Tan RG, Beets GL (2010). Long-term outcome in patients with a pathological complete response after chemoradiation for rectal cancer: a pooled analysis of individual patient data. Lancet Oncol.

[7] Mohiuddin M, Hayne M, Regine WF, Hanna N, Hagihara PF, McGrath P, Marks GM (2000). Prognostic significance of postchemoradiation stage following preoperative chemotherapy and radiation for advanced/recurrent rectal cancers. Int J Radiat Oncol Biol Phys.

[8] Sadahiro S, Suzuki T, Tanaka A, Okada K, Saito G, Kamijo A, Nagase H (2016). Increase in gene expression of TYMP, DPYD and HIF1A are associated with response to preoperative chemoradiotherapy including S-1 or UFT for rectal cancer. Anticancer Res.

[9] Wolthuis AM, Penninckx F, Haustermans K, Ectors N, Van Cutsem E, D’Hoore A (2011). Outcome standards for an organ preservation strategy in stage II and III rectal adenocarcinoma after neoadjuvant chemoradiation. Ann Surg Oncol.

[10] Garcia-Aguilar J, Chow OS, Smith DD, Marcet JE, Cataldo PA, Varma MG, Kumar AS, Oommen S, Coutsoftides T, Hunt SR, Stamos MJ, Ternent CA, Herzig DO, Fichera A, Polite BN, Dietz DW, Patil S, Avila K (2015). Effect of adding mFOLFOX6 after neoadjuvant chemoradiation in locally advanced rectal cancer: a multicentre, phase 2 trial. Lancet Oncol.

[11] Berger SH, Hakala MT (1984). Relationship of dUMP and free FdUMP pools to inhibition of thymidylate synthase by 5-fluorouracil. Mol Pharmacol.

[12] Houghton JA, Williams LG, Radparvar S, Houghton PJ (1988). Characterization of the pools of 5,10-methylenetetrahydrofolates and tetrahydrofolates in xenografts of human colon adenocarcinoma. Cancer Res.

[13] Morgan RG (1989). Leucovorin enhancement of the effects of the fluoropyrimidines on thymidylate synthase. Cancer.

[14] Carmichael J, Popiela T, Radstone D, Falk S, Borner M, Oza A, Skovsgaard T, Munier S, Martin C (2002). Randomized comparative study of tegafur/uracil and oral leucovorin versus parenteral fluorouracil and leucovorin in patients with previously untreated metastatic colorectal cancer. J Clin Oncol.

[15] Douillard JY, Hoff PM, Skillings JR, Eisenberg P, Davidson N, Harper P, Vincent MD, Lembersky BC, Thompson S, Maniero A, Benner SE (2002). Multicenter phase III study of uracil/tegafur and oral leucovorin versus fluorouracil and leucovorin in patients with previously untreated metastatic colorectal cancer. J Clin Oncol.

[16] Sadahiro S, Tsuchiya T, Sasaki K, Kondo K, Katsumata K, Nishimura G, Kakeji Y, Baba H, Sato S, Koda K, Yamaguchi Y, Morita T, Matsuoka J, Usuki H, Hamada C, Kodaira S (2015). Randomized phase III trial of treatment duration for oral uracil and tegafur plus leucovorin as adjuvant chemotherapy for patients with stage IIB/III colon cancer: final results of JFMC33-0502. Ann Oncol.

[17] Sadahiro S, Suzuki T, Tanaka A, Okada K, Saito G, Kamijo A, Akiba T, Kawada S (2015). Phase II study of preoperative concurrent chemoradiotherapy with S-1 plus bevacizumab for locally advanced resectable rectal adenocarcinoma. Oncology.

[18] Sadahiro S, Suzuki T, Tanaka A, Okada K, Kamijo A, Murayama C, Akiba T, Nakayama Y (2011). Phase I/II study of preoperative concurrent chemoradiotherapy with S-1 for locally advanced, resectable rectal adenocarcinoma. Oncology.

[19] Suzuki T, Sadahiro S, Tanaka A, Okada K, Saito G, Kamijo A, Akiba T, Kawada S (2014). Relationship between histologic response and the degree of tumor shrinkage after chemoradiotherapy in patients with locally advanced rectal cancer. J Surg Oncol.

[20] Suzuki T, Sadahiro S, Tanaka A, Okada K, Kamata H, Kamijo A, Murayama C, Akiba T, Kawada S (2013). Biopsy specimens obtained 7 days after starting chemoradiotherapy (CRT) provide reliable predictors of response to CRT for rectal cancer. Int J Radiat Oncol Biol Phys.

[21] Sadahiro S, Suzuki T, Ishikawa K, Fukasawa M, Saguchi T, Yasuda S, Makuuchi H, Murayama C, Ohizumi Y (2004). Preoperative radio/chemo-radiotherapy in combination with intraoperative radiotherapy for T3-4Nx rectal cancer. Eur J Surg Oncol.

[22] Sadahiro S, Suzuki T, Tanaka A, Okada K, Nagase H, Uchida J (2012). Association of right-sided tumors with high thymidine phosphorylase gene expression levels and the response to oral uracil and tegafur/leucovorin chemotherapy among patients with colorectal cancer. Cancer Chemother Pharmacol.

[23] Sadahiro S, Suzuki T, Maeda Y, Tanaka A, Ogoshi K, Kamijo A, Murayama C, Tsukioka S, Sakamoto E, Fukui Y, Oka T (2010). Molecular determinants of folate levels after leucovorin administration in colorectal cancer. Cancer Chemother Pharmacol.

[24] Sadahiro S, Suzuki T, Tanaka A, Okada K, Kamijo A, Nagase H, Uchida J (2013). Reduction in gamma-glutamyl hydrolase expression is associated with response to uracil and tegafur/leucovorin chemotherapy in patients with colorectal cancer. Anticancer Res.

[25] Mandard AM, Dalibard F, Mandard JC, Marnay J, Henry-Amar M, Petiot JF, Roussel A, Jacob JH, Segol P, Samama G (1994). Pathologic assessment of tumor regression after preoperative chemoradiotherapy of esophageal carcinoma. Clinicopathologic correlations. Cancer.

[26] Suzuki T, Sadahiro S, Fukasawa M, Ishikawa K, Kamijo A, Yasuda S, Makuuchi H, Ohizumi Y, Murayama C (2004). Predictive factors of tumor shrinkage and histological regression in patients who received preoperative radiotherapy for rectal cancer. Jpn J Clin Oncol.

[27] Yeo SG, Kim DY, Kim TH, Jung KH, Hong YS, Chang HJ, Park JW, Lim SB, Choi HS, Jeong SY (2010). Tumor volume reduction rate measured by magnetic resonance volumetry correlated with pathologic tumor response of preoperative chemoradiotherapy for rectal cancer. Int J Radiat Oncol Biol Phys.

[28] Eisenhauer EA, Therasse P, Bogaerts J, Schwartz LH, Sargent D, Ford R, Dancey J, Arbuck S, Gwyther S, Mooney M, Rubinstein L, Shankar L, Dodd L, Kaplan R, Lacombe D, Verweij J (2009). New response evaluation criteria in solid tumours: revised RECIST guideline (version 1.1). Eur J Cancer.

[29] Mori R, Wang Q, Danenberg KD, Pinski JK, Danenberg PV (2008). Both beta-actin and GAPDH are useful reference genes for normalization of quantitative RT-PCR in human FFPE tissue samples of prostate cancer. Prostate.

[30] Kreuzer KA, Lass U, Landt O, Nitsche A, Laser J, Ellerbrok H, Pauli G, Huhn D, Schmidt CA (1999). Highly sensitive and specific fluorescence reverse transcription-PCR assay for the pseudogene-free detection of beta-actin transcripts as quantitative reference. Clin Chem.

[31] Huntington CR, Boselli D, Symanowski J, Hill JS, Crimaldi A, Salo JC (2016). Optimal timing of surgical resection after radiation in locally advanced rectal adenocarcinoma: an analysis of the national cancer database. Ann Surg Oncol.

[32] Rombouts AJ, Hugen N, Elferink MA, Nagtegaal ID, de Wilt JH (2016). Treatment interval between neoadjuvant chemoradiotherapy and surgery in rectal cancer patients: a population-based study. Ann Surg Oncol.

[33] Sun Z, Adam MA, Kim J, Shenoi M, Migaly J, Mantyh CR (2016). Optimal timing to surgery after neoadjuvant chemoradiotherapy for locally advanced rectal cancer. J Am Coll Surg.

[34] Lefevre JH, Mineur L, Kotti S, Rullier E, Rouanet P, de Chaisemartin C, Meunier B, Mehrdad J, Cotte E, Desrame J, Karoui M, Benoist S, Kirzin S, Berger A, Panis Y, Piessen G, Saudemont A, Prudhomme M, Peschaud F, Dubois A, Loriau J, Tuech JJ, Meurette G, Lupinacci R, Goasgen N, Parc Y, Simon T, Tiret E (2016). Effect of interval (7 or 11 weeks) between neoadjuvant radiochemotherapy and surgery on complete pathologic response in rectal cancer: a multicenter, randomized, controlled trial (GRECCAR-6). J Clin Oncol.

[35] Suh JR, Herbig AK, Stover PJ (2001). New perspectives on folate catabolism. Annu Rev Nutr.

[36] Schneider E, Ryan TJ (2006) Gamma-glutamyl hydrolase and drug resistance. Clin Chim Acta Int J Clin Chem 374(1–2):25–32. doi:10.1016/j.cca.2006.05.044 10.1016/j.cca.2006.05.044 16859665

[37] Sakamoto E, Tsukioka S, Oie S, Kobunai T, Tsujimoto H, Sakamoto K, Okayama Y, Sugimoto Y, Oka T, Fukushima M (2008). Folylpolyglutamate synthase and gamma-glutamyl hydrolase regulate leucovorin-enhanced 5-fluorouracil anticancer activity. Biochem Biophys Res Commun.

[38] Fox JT, Stover PJ (2008). Folate-mediated one-carbon metabolism. Vitam Horm.

[39] Tsukihara H, Tsunekuni K, Takechi T (2016). Folic acid-metabolizing enzymes regulate the antitumor effect of 5-fluoro-2′-deoxyuridine in colorectal cancer cell lines. PloS one.

[40] Rahman L, Voeller D, Rahman M, Lipkowitz S, Allegra C, Barrett JC, Kaye FJ, Zajac-Kaye M (2004). Thymidylate synthase as an oncogene: a novel role for an essential DNA synthesis enzyme. Cancer cell.

[41] Davis ST, Berger SH (1993). Variation in human thymidylate synthase is associated with resistance to 5-fluoro-2′-deoxyuridine. Mol Pharmacol.

[42] Johnston PG, Lenz HJ, Leichman CG, Danenberg KD, Allegra CJ, Danenberg PV, Leichman L (1995). Thymidylate synthase gene and protein expression correlate and are associated with response to 5-fluorouracil in human colorectal and gastric tumors. Cancer Res.

[43] Peters GJ, van der Wilt CL, van Groeningen CJ, Smid K, Meijer S, Pinedo HM (1994). Thymidylate synthase inhibition after administration of fluorouracil with or without leucovorin in colon cancer patients: implications for treatment with fluorouracil. J Clin Oncol.

[44] Moeller BJ, Dewhirst MW (2006). HIF-1 and tumour radiosensitivity. Br J Cancer.

[45] Toiyama Y, Inoue Y, Saigusa S, Okugawa Y, Yokoe T, Tanaka K, Miki C, Kusunoki M (2010). Gene expression profiles of epidermal growth factor receptor, vascular endothelial growth factor and hypoxia-inducible factor-1 with special reference to local responsiveness to neoadjuvant chemoradiotherapy and disease recurrence after rectal cancer surgery. Clin Oncol (R Coll Radiol).

